# Childhood Depression, Hopelessness, and Suicidal Attempt Predict Earlier Tobacco and Marijuana Use Initiation During Adolescence

**DOI:** 10.31586/ojms.2025.1181

**Published:** 2025-02-11

**Authors:** Shervin Assari, Babak Najand, Payam Sheikhattari

**Affiliations:** 1 Department of Internal Medicine, Charles R Drew University of Medicine and Science, Los Angeles, CA, USA; 2 Department of Urban Public Health, Charles R Drew University of Medicine and Science, Los Angeles, CA, USA; 3 Marginalization-related Diminished Returns, Los Angeles, CA, USA; 4 Center for Urban Health Disparities Research and Innovation, Morgan State University, Baltimore, MD, USA; 5 The Prevention Sciences Research Center, School of Community Health and Policy, Morgan State University, Baltimore, MD, USA; 6 Department of Public and Allied Health, School of Community Health and Policy, Morgan State University, Baltimore, MD, USA

**Keywords:** Adolescent Substance Use, Childhood Depression, Hopelessness, Suicidal Ideation, Tobacco Use, Marijuana Use, Mental Health, Longitudinal Research, Socioeconomic Disparities, Racial Disparities, Emotional Regulation, Adolescence

## Abstract

**Background:**

Emotional problems have been associated with substance use, yet longitudinal research examining this relationship during childhood and adolescence in large, diverse, community-based samples remains limited.

**Aims:**

This study investigates the prospective associations between three emotional problems—hopelessness, depression, and suicide attempts—before ages 9–10 and the subsequent initiation of tobacco and marijuana use before ages 14–15, using data from the Adolescent Brain Cognitive Development (ABCD) study.

**Methods:**

Data from the ABCD study were analyzed. Baseline emotional problems, including hopelessness, depression, and suicide attempts, were assessed at ages 9–10 through structured parent interviews. Substance use outcomes (tobacco and marijuana initiation) were tracked from baseline to follow-up at ages 14–15 using structured self-report measures. Structural Equation Modeling (SEM) was employed to assess the predictive roles of these early-life emotional problems, controlling for potential confounders such as demographic factors and family and neighborhood socioeconomic status.

**Results:**

Baseline hopelessness, depression, and suicide attempts at ages 9–10 were significant predictors of tobacco and marijuana use initiation at ages 14–15. These associations remained robust after adjusting for confounders, indicating the independent effects of early emotional problems on adolescent substance use initiation.

**Conclusions:**

Emotional problems in early childhood, including hopelessness, depression, and suicidal behavior, are critical predictors of substance use initiation during adolescence. These findings underscore the importance of early identification and targeted mental health interventions to reduce the risk of substance use among vulnerable youth.

## 1. Introduction

Substance use during adolescence presents critical public health challenges, with enduring implications for health and well-being [1-4]. Tobacco and marijuana, two of the most commonly used substances among adolescents, have been linked to adverse physical, cognitive, and mental health outcomes [4-9]. Identifying early predictors of substance use initiation is essential for guiding prevention efforts and designing interventions that address these pressing concerns.

Depression, hopelessness, and suicidal ideation are among the most common indicators of poor mental health during childhood, often contributing to negative developmental trajectories [10-13]. Previous research has suggested that childhood depression and associated hopelessness and suicidal ideation may heighten vulnerability to substance use [14-16]. However, the specific effects of these mental health challenges on adolescent tobacco and marijuana use remain insufficiently understood. Moreover, several gaps in the existing literature necessitate further investigation. First, prior studies have often been limited in diversity, with findings primarily derived from White, middleclass samples, raising questions about the generalizability of results to other racial, ethnic, and socioeconomic groups. Additionally, much of the evidence stems from clinical samples or cross-sectional studies, which provide limited insight into the temporal relationship between childhood mental health challenges and adolescent substance use initiation. Finally, the distinct and combined effects of depression, hopelessness, and suicidality on future substance use remain unclear, particularly when accounting for the interplay between these factors. Clarifying whether these constructs exert independent or overlapping influences on substance use initiation is a key priority for advancing the field.

Gender differences may also play a critical role in the relationship between childhood mental health and adolescent substance use [17]. Existing research highlights that boys and girls may experience and respond to depression, hopelessness, and suicidality in distinct ways, influenced by differences in coping strategies, socialization patterns, and biological factors [18-20]. For example, girls are more likely to internalize emotional distress, potentially increasing their risk of substance use as a coping mechanism, whereas boys are more likely to externalize distress through risk-taking behaviors, including substance use [21-23]. Investigating these gender differences is essential for tailoring prevention and intervention efforts to meet the specific needs of both boys and girls.

This study leverages the longitudinal data from the Adolescent Brain Cognitive Development (ABCD) [24-35] study to examine the relationship between childhood depression and subsequent adolescent substance use. Specifically, it evaluates whether depression at ages 9–10 predicts tobacco and marijuana use at ages 14–15, while accounting for potential confounding variables. Additionally, this study explores whether the relationship between childhood depression and substance use differs by gender, providing critical insights into gender-specific pathways to substance use initiation.

## 2. Methods

### Design

This study utilized data from the Adolescent Brain Cognitive Development (ABCD) [24-35] study, a longitudinal investigation of adolescent development and health across the United States. The ABCD study follows a cohort of children from ages 9–10 into adulthood, collecting extensive data on their biological, psychological, and social development. This analysis focuses on identifying predictors of tobacco and marijuana use during adolescence and understanding how childhood mental health indicators such as depression, hopelessness, and suicidality influence substance use initiation.

### Sampling

The ABCD study employed a multi-stage probability sampling design to recruit a demographically diverse sample. Participants were selected from 21 study sites across the United States to ensure geographic, socioeconomic, and racial/ethnic diversity. Recruitment was primarily conducted through public and private schools, using stratification to reflect the sociodemographic composition of the U.S. population. This sampling approach enhances the generalizability of the findings.

### Sample

The analytic sample for this study included children aged 9–10 at baseline (Wave 1) who had completed measures of mental health and substance use initiation by ages 14–15 (Wave 5). Participants with missing data on key variables, including depression, hopelessness, suicidality, and substance use outcomes, were excluded from the analysis. The final sample consisted of approximately [insert number of participants] adolescents, with balanced representation across gender and diverse racial/ethnic and socioeconomic backgrounds.

### Measures

#### 1. Mental Health Indicators

##### Depression:

Assessed using the Child Behavior Checklist (CBCL), which measures symptoms of depression reported by caregivers.

##### Hopelessness:

Evaluated using the Hopelessness Scale for Children (HSC), a self-report measure that captures cognitive and emotional dimensions of hopelessness.

##### Suicidality:

Measured through child-reported items on the Kiddie Schedule for Affective Disorders and Schizophrenia (K-SADS) that assess thoughts of self-harm or suicidal ideation.

#### 2. Substance Use Outcomes

##### Tobacco Use:

Self-reported initiation of tobacco products (e.g., cigarettes, e-cigarettes) by ages 14–15.

##### Marijuana Use:

Self-reported initiation of marijuana use (including vaping or smoking) by ages 14–15.

#### 3. Covariates

Sociodemographic factors, including gender, race/ethnicity, household income, and parental education, were included to control for potential confounders.

### Institutional Review Board (IRB)

The ABCD study received approval from the institutional review boards at all participating study sites, including the University of California, San Diego (UCSD), ensuring ethical compliance in data collection and participant protections. Informed consent was obtained from parents or legal guardians, and assent was obtained from child participants before their involvement. The secondary analysis of de-identified data for this study was exempt from further IRB review under U.S. regulations governing research with existing data [IRB Net Number: 1811107-2].

### Analysis

Descriptive statistics were calculated to summarize the sample's demographic characteristics and the prevalence of mental health indicators and substance use. Structural Equation Models (SEMs) [36-40] were employed to examine the associations between childhood depression, hopelessness, and suicidality at ages 9–10 and the initiation of tobacco and marijuana use by ages 14–15. First, models were run in the pooled sample. Next, multi-group models were conducted, stratified by gender, to assess potential gender differences in these relationships. Analyses were performed using Stata 18.0 [41-43], with statistical significance set at p < 0.05. Model fit was evaluated using RMSEA and CFI indices.

## 3. Results

The results of the Structural Equation Model (SEM) examining the predictors of tobacco and marijuana use in the pooled sample are summarized in Table 1. Below, we present the key findings for each substance.

### 3.1. Subsequent Tobacco Use Initiation in Adolescence

Major Depressive Disorder (MDD) during childhood was positively associated with tobacco use (B = 0.030, SE = 0.011, 95% CI [0.008, 0.051], p = 0.007). Suicide attempt and hopelessness during childhood were both significant predictors of higher subsequent tobacco use initiation in adolescence (B = 0.063, SE = 0.014, 95% CI [0.035, 0.091], p < 0.001; B = 0.046, SE = 0.015, 95% CI [0.016, 0.076], p = 0.003, respectively). Age was positively associated with tobacco use (B = 0.076, SE = 0.009, 95% CI [0.058, 0.094], p < 0.001), indicating increased use with age. Gender (male) showed a marginally non-significant negative association with tobacco use (B = −0.017, SE = 0.009, 95% CI [−0.035, 0.001], p = 0.058). Family Income did not significantly predict tobacco use (B = −0.015, SE = 0.015, 95% CI [−0.045, 0.014], p = 0.305). Parental Education was negatively associated with tobacco use (B = −0.028, SE = 0.013, 95% CI [−0.052, −0.003], p = 0.028), suggesting a protective effect. Living in a married household was strongly associated with lower tobacco use (B = −0.054, SE = 0.011, 95% CI [−0.076, −0.032], p < 0.001). Neighborhood Median Income showed a marginally non-significant positive trend (B = 0.020, SE = 0.011, 95% CI [−0.001, 0.042], p = 0.061). Race/ethnicity analyses revealed that Black and Asian individuals were less likely to use tobacco compared to the reference group (B = −0.045, SE = 0.011, 95% CI [−0.067, −0.023], p < 0.001; B = −0.027, SE = 0.009, 95% CI [−0.045, −0.009], p = 0.003). Latino ethnicity did not show a significant effect (B = −0.001, SE = 0.011, 95% CI [−0.022, 0.020], p = 0.905). Other race/ethnicity categories showed no significant associations.

### 3.2. Subsequent Marijuana Use Initiation in Adolescence

Childhood MDD had a positive effect on subsequent marijuana use initiation in adolescence (B = 0.024, SE = 0.011, 95% CI [0.002, 0.045], p = 0.032), as did suicide attempt (B = 0.064, SE = 0.014, 95% CI [0.037, 0.092], p < 0.001) and childhood hopelessness (B = 0.031, SE = 0.015, 95% CI [0.001, 0.060], p = 0.041). Age showed a positive association with marijuana use (B = 0.078, SE = 0.009, 95% CI [0.060, 0.096], p < 0.001). Gender (male) did not significantly predict marijuana use (B = 0.001, SE = 0.009, 95% CI [−0.017, 0.019], p = 0.899). Family income and parental education both had significant negative associations with marijuana use (B = −0.031, SE = 0.015, 95% CI [−0.060, −0.001], p = 0.040; B = −0.025, SE = 0.012, 95% CI [−0.049, 0.000], p = 0.047, respectively).Living in a married household in childhood was associated with reduced subsequent marijuana use initiation during adolescence (B = −0.050, SE = 0.011, 95% CI [−0.073, −0.028], p < 0.001). Neighborhood median income during childhood was not a significant predictor of subsequent marijuana use (B = 0.004, SE = 0.011, 95% CI [−0.017, 0.025], p = 0.698). Similar to subsequent tobacco use initiation, Black and Asian individuals were less likely to use marijuana compared to the reference group (B = −0.031, SE = 0.011, 95% CI [−0.054, −0.009], p = 0.006; B = −0.019, SE = 0.009, 95% CI [−0.037, −0.001], p = 0.039). Latino ethnicity and other race/ethnicity categories were not significant predictors (B = −0.010, SE = 0.011, 95% CI [−0.031, 0.011], p = 0.334; B = −0.004, SE = 0.010, 95% CI [−0.023, 0.015], p = 0.655).

### 3.3. Gender Differences in Predictors of Tobacco and Marijuana Use

#### 3.3.1. Subsequent Tobacco Use Initiation in Adolescence

Major Depressive Disorder (MDD) was a significant predictor for females (B = 0.049, SE = 0.016, 95% CI [0.018, 0.081], p = 0.002) but not for males (B = 0.015, SE = 0.015, p = 0.338). Suicide Attempt and Hopelessness were strong predictors for females (B = 0.104, SE = 0.020, p < 0.001; B = 0.087, SE = 0.022, p < 0.001, respectively), but non-significant for males (B = 0.023, SE = 0.021, p = 0.272; B = 0.022, SE = 0.021, p = 0.306, respectively). Age was positively associated with tobacco use for both males (B = 0.067, SE = 0.013, 95% CI [0.042, 0.092], p < 0.001) and females (B = 0.084, SE = 0.013, 95% CI [0.058, 0.110], p < 0.001). Family Income was not significantly associated with tobacco use in either gender (B = −0.031, SE = 0.021, p = 0.134 for males and B = 0.007, SE = 0.022, p = 0.754 for females). Parental Education showed a protective effect for males (B = −0.040, SE = 0.017, 95% CI [−0.073, −0.006], p = 0.021) but not for females (B = −0.019, SE = 0.018, p = 0.307). Living in a married Household had a stronger protective effect for females (B = −0.084, SE = 0.016, 95% CI [−0.116, −0.052], p < 0.001) compared to males, where it was non-significant (B = −0.025, SE = 0.016, p = 0.116). Neighborhood Median Income showed a positive association for females (B = 0.034, SE = 0.016, 95% CI [0.003, 0.065], p = 0.032), whereas it was non-significant for males (B = 0.007, SE = 0.015, p = 0.654). Race/Ethnicity effects were observed for both genders. Black individuals showed lower tobacco use for both females (B = −0.050, SE = 0.017, p = 0.003) and males (B = −0.040, SE = 0.016, p = 0.010). Asian individuals had lower tobacco use among females (B = −0.035, SE = 0.014, p = 0.010), but the effect was non-significant for males (B = −0.021, SE = 0.013, p = 0.101).

#### 3.3.2. Subsequent Marijuana Use Initiation in Adolescence

MDD was a significant predictor for females (B = 0.037, SE = 0.016, 95% CI [0.006, 0.068], p = 0.020), but not for males (B = 0.015, SE = 0.015, p = 0.317). Suicide Attempt was a strong predictor of marijuana use for females (B = 0.127, SE = 0.019, p < 0.001) but not significant for males (B = 0.004, SE = 0.020, p = 0.836). Hopelessness was marginally significant for females (B = 0.042, SE = 0.021, p = 0.051) and non-significant for males (B = 0.029, SE = 0.021, p = 0.166). Age was a significant predictor for both males (B = 0.084, SE = 0.013, 95% CI [0.059, 0.109], p < 0.001) and females (B = 0.071, SE = 0.013, 95% CI [0.045, 0.098], p < 0.001). Family Income was negatively associated with marijuana use for males (B = −0.052, SE = 0.020, 95% CI [−0.092, −0.011], p = 0.012) but showed no association for females (B = 0.002, SE = 0.022, p = 0.911). Parental Education was not significantly associated with marijuana use for either gender, though a trend toward a protective effect was observed for males (B = −0.030, SE = 0.017, p = 0.076). Living in a married Household was a strong protective factor for females (B = −0.089, SE = 0.016, 95% CI [−0.121, −0.056], p < 0.001) but not significant for males (B = −0.019, SE = 0.016, p = 0.223). Race/Ethnicity effects were observed primarily among males. Black males had lower marijuana use (B = −0.036, SE = 0.016, p = 0.022), while no significant effects were observed for females across race/ethnicity categories.

### 3.4. Summary of Gender Differences

The results highlight significant gender differences in the predictors of tobacco and marijuana use. Psychological factors, including MDD, suicide attempts, and hopelessness, were stronger predictors for females, while socioeconomic factors such as family income and parental education were more relevant for males. Additionally, the protective effects of living in a married household were more pronounced among females, emphasizing the importance of considering gender-specific mechanisms when addressing substance use behaviors.

## 4. Discussion

The primary aim of this study was to investigate whether childhood depression, hopelessness, and suicidality at ages 9–10 predict the subsequent initiation of tobacco and marijuana use during early adolescence. Additionally, this study explored whether these associations differ by gender. Utilizing data from the Adolescent Brain Cognitive Development (ABCD) study, we sought to address significant gaps in the literature, which often relies on clinical samples, cross-sectional designs, and lacks consideration of the distinct roles of hopelessness, suicidality, and gender-specific pathways.

This study demonstrates that childhood depression, hopelessness, and suicidality are significant predictors of both tobacco and marijuana use initiation during adolescence. These findings expand on prior research by highlighting the multifaceted impact of mental health challenges on substance use. Our results suggest that these mental health indicators independently impair self-regulation and coping mechanisms, increasing the likelihood of adolescents turning to substance use as a maladaptive strategy for managing emotional distress. Neurobiological changes associated with depression and hopelessness, such as dysregulation of reward processing, may further heighten sensitivity to the reinforcing effects of substances like tobacco and marijuana.

Our analysis also reveals nuanced gender differences in the relationship between childhood mental health challenges and substance use initiation. While boys and girls may share some common pathways, differences in coping mechanisms, emotional regulation, and socialization patterns are critical. For example, girls may be more prone to internalizing emotional distress, increasing their likelihood of using substances to selfmedicate. Boys, conversely, may externalize their distress, engaging in risk-taking behaviors, including substance use. Understanding these gender-specific pathways is essential for tailoring prevention and intervention strategies to meet the unique needs of both genders.

Childhood depression, hopelessness, and suicidality may contribute to substance use through interconnected psychological, neurobiological, and social mechanisms. Psychologically, depressive symptoms impair emotional regulation, making it more likely that adolescents will rely on substances as a way to cope with distress. Neurobiologically, depression disrupts the functioning of reward pathways, altering sensitivity to the reinforcing effects of substances such as tobacco and marijuana. Social withdrawal, which is commonly associated with depression, may further exacerbate these risks by reducing access to protective influences, such as supportive friendships, and increasing susceptibility to negative peer influences. These mechanisms underscore the complexity of the relationship between childhood mental health and substance use, as well as the need for multifaceted interventions that address both the psychological and neurobiological roots of these outcomes while fostering strong social support networks.

The findings emphasize the urgent need for policies that prioritize early mental health screening and intervention. Universal depression screenings in pediatric healthcare and school-based wellness programs could help identify at-risk children earlier. Equitable access to mental health resources is critical, particularly for populations disproportionately affected by systemic inequities. Addressing broader social determinants, such as poverty, housing instability, and neighborhood safety, could further mitigate childhood mental health challenges and their downstream effects. Expanding funding for comprehensive mental health services and integrating these resources into public health frameworks are essential advocacy efforts to reduce the prevalence of childhood mental health challenges and subsequent substance use.

Preventing substance use among youth with a history of depression, hopelessness, or suicidality requires multi-level approaches. School-based programs that teach emotional regulation, stress management, and coping skills can help mitigate the risks associated with these mental health challenges. Family-focused interventions that strengthen parent-child communication and relationships may provide an additional protective buffer against substance use initiation. Community-based initiatives, such as mentoring programs and recreational activities, can foster protective environments that reduce isolation and promote resilience. Individual-level interventions, including evidence-based treatments like cognitive-behavioral therapy (CBT), should be made widely accessible, potentially through innovative platforms like telehealth, which can help overcome traditional barriers to care.

Structural inequities in mental health care access, parental mental health literacy, and stigma remain substantial barriers to early diagnosis and treatment of childhood mental health challenges. Integrating school-based screenings with parental involvement and referrals to accessible mental health services could help mitigate the heightened risk of substance use in children with depression, hopelessness, or suicidality. For marginalized communities, systemic barriers such as poverty and racism further amplify these risks. Addressing these disparities requires investments in community resources, including high-quality mental health care, education, and neighborhood infrastructure. Comprehensive policies aimed at reducing socioeconomic disadvantages and promoting equity are essential for improving mental health outcomes and reducing the risks of substance use.

### 4.1. Limitations

This study has several limitations that should be considered when interpreting its findings. First, although the Adolescent Brain Cognitive Development (ABCD) study provides a large and diverse community-based sample, the reliance on self-reports for both substance use initiation and emotional problems introduces the possibility of measurement bias. This bias could stem from recall errors or social desirability influences. Furthermore, the use of parent-reported data for emotional problems at baseline may lead to discrepancies, as the interviewed parent may not always be the primary caregiver or the individual most attuned to the child’s mental health challenges. Parents may also be unaware of their child’s internal experiences, particularly regarding depression, hopelessness, and suicidality, which are inherently subjective constructs.

Second, the study’s observational design limits the ability to draw causal inferences about the relationship between emotional problems and substance use initiation. While the longitudinal design establishes temporal order and adjustments were made for potential confounders such as socioeconomic and demographic factors, the findings may still be influenced by unmeasured confounders. These could include genetic predispositions, peer influences, and broader environmental factors, all of which may play a role in shaping the observed associations. The specific mechanisms linking hopelessness, depression, and suicidality to substance use initiation remain unclear, highlighting the need for further investigation into these pathways.

Third, the analysis focused on overall associations and did not test for potential heterogeneity in these relationships across subgroups based on race, ethnicity, geographic location, or urbanity. This limitation restricts the ability to understand how these associations may differ across diverse contexts, which is critical for tailoring interventions to specific populations.

Fourth, emotional problems were measured at a single time point (ages 9–10), limiting the ability to assess changes in these emotional challenges over time and their evolving impact on substance use. Emotional trajectories can vary significantly among children, and capturing these changes would provide more nuanced insights into the dynamic relationship between mental health and substance use initiation.

Fifth, while the ABCD sample is national and diverse, it was not randomly selected, which may limit the generalizability of the findings. Specific cultural, racial/ethnic, and socioeconomic nuances that influence the relationship between emotional problems and substance use may not have been fully captured. Future research should more deeply explore these interactions, particularly among underrepresented and marginalized populations.

Finally, this study focused exclusively on tobacco and marijuana initiation as the primary outcomes, without considering polysubstance use or the progression to more frequent or problematic patterns of use. Understanding whether early emotional problems contribute to more severe or chronic substance use remains an important avenue for future research.

Despite these limitations, the study offers valuable longitudinal evidence of the predictive role of early emotional problems in adolescent substance use initiation. It underscores the importance of early identification and targeted intervention strategies and highlights key areas for further exploration in both research and practice.

### 4.2. Future research

Future research should explore the distinct and combined effects of depression, hopelessness, and suicidality on substance use. Longitudinal studies can provide deeper insights into protective factors and the mechanisms that buffer against substance use risks, particularly among diverse and underserved populations. Understanding how structural inequities influence these relationships is vital for designing targeted interventions and policies that address these disparities.

## 5. Conclusion

In conclusion, this study underscores the significant role of childhood depression, hopelessness, and suicidality in predicting the initiation of tobacco and marijuana use during adolescence. By addressing the psychological, neurobiological, and structural factors contributing to these outcomes, we can develop comprehensive strategies to mitigate risks and promote healthier trajectories for all children, particularly those from marginalized communities. Investing in early mental health interventions, equitable policies, and multi-level approaches is critical for breaking the cycle of mental health challenges and substance use.

## Figures and Tables

**Figure 1.**
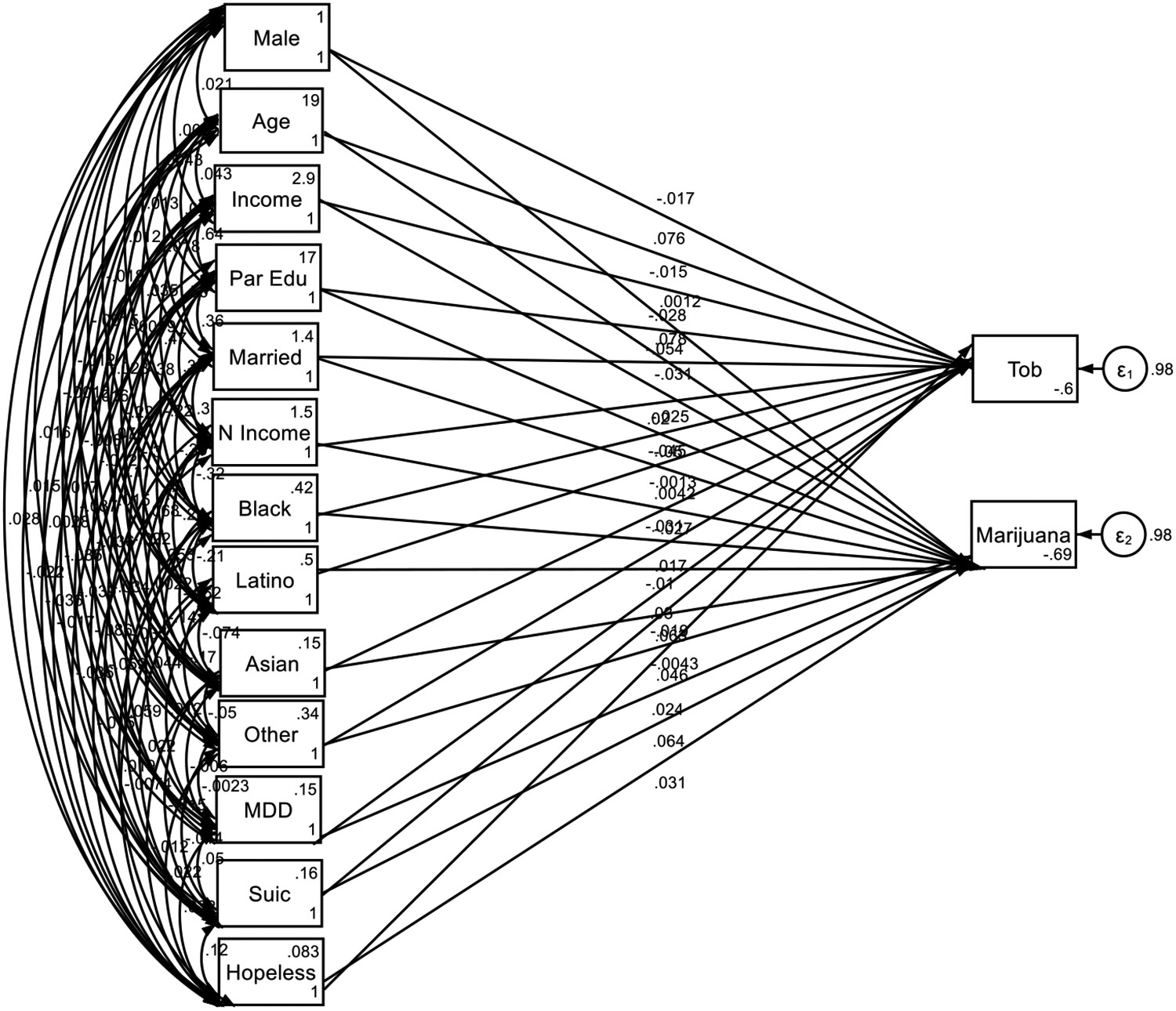
Structural Equation Model (SEM), Overall

**Figure 2.**
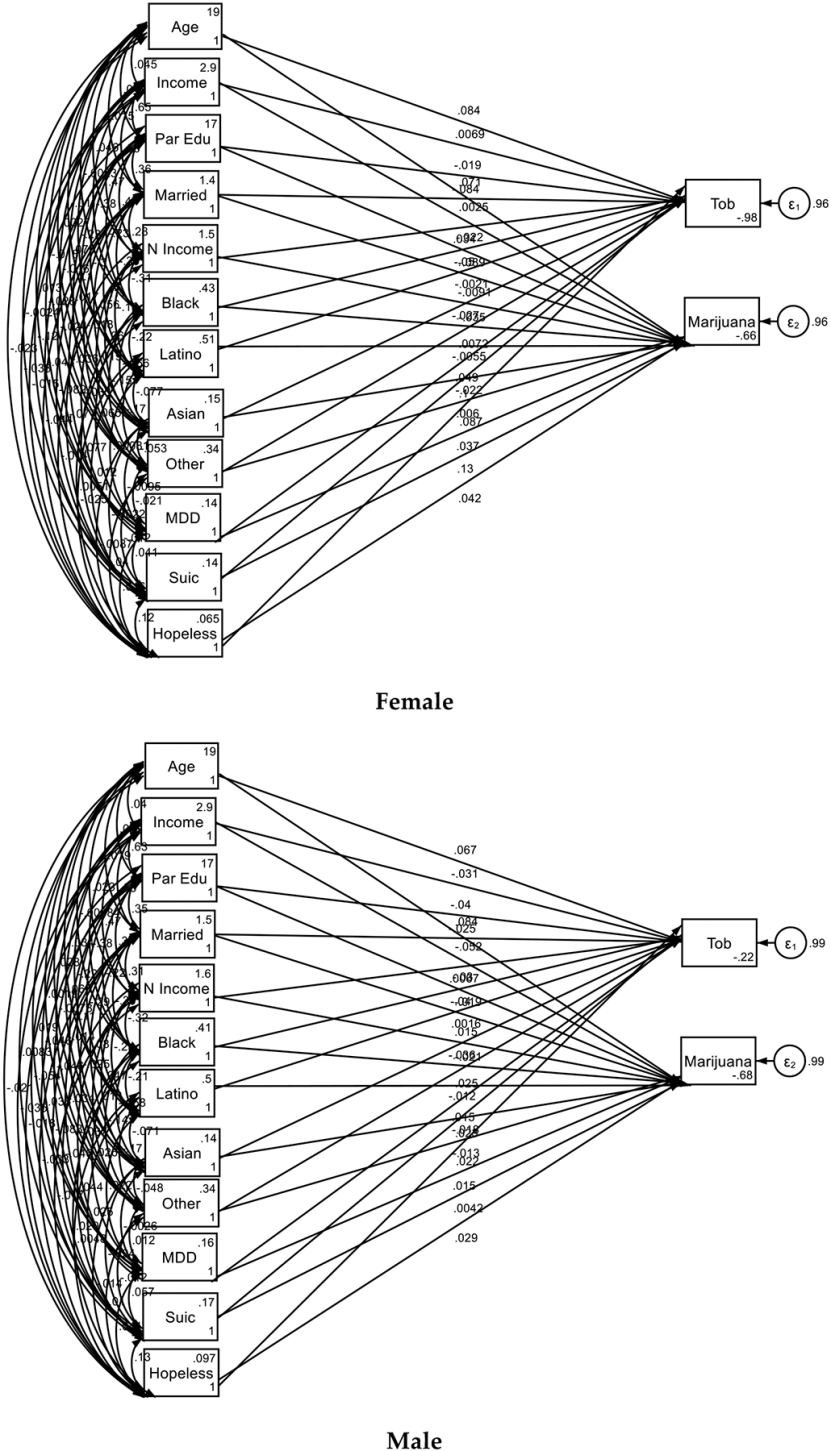
Multi-group Structural Equation Models (SEMs), by Gender

**Table 1.** Summary of Structural Equation Model (SEM), Overall

Independent Variable	Dependent Variable	B	SE	95%	CI	P
Age (Years)	Tobacco Use	0.076	0.009	0.058	0.094	< 0.001
Gender (Male)	Tobacco Use	−0.017	0.009	−0.035	0.001	0.058
Family Income	Tobacco Use	−0.015	0.015	−0.045	0.014	0.305
Parental Education	Tobacco Use	−0.028	0.013	−0.052	−0.003	0.028
Married Household	Tobacco Use	−0.054	0.011	−0.076	−0.032	< 0.001
Neighborhood Median Income/ 50000	Tobacco Use	0.020	0.011	−0.001	0.042	0.061
Major Depressive Disorder	Tobacco Use	0.030	0.011	0.008	0.051	0.007
Suicide Attempt	Tobacco Use	0.063	0.014	0.035	0.091	< 0.001
Hopeless	Tobacco Use	0.046	0.015	0.016	0.076	0.003
Race/Ethnicity (Black)	Tobacco Use	−0.045	0.011	−0.067	−0.023	< 0.001
Race/Ethnicity (Latino)	Tobacco Use	−0.001	0.011	−0.022	0.020	0.905
Race/Ethnicity (Asian)	Tobacco Use	−0.027	0.009	−0.045	−0.009	0.003
Race/Ethnicity (Other)	Tobacco Use	0.017	0.010	−0.002	0.035	0.087
Intercept	Tobacco Use	−0.597	0.261	−1.110	−0.085	0.022


Age (Years)	Marijuana Use	0.078	0.009	0.060	0.096	< 0.001
Gender (Male)	Marijuana Use	0.001	0.009	−0.017	0.019	0.899
Family Income	Marijuana Use	−0.031	0.015	−0.060	−0.001	0.040
Parental Education	Marijuana Use	−0.025	0.012	−0.049	0.000	0.047
Married Household	Marijuana Use	−0.050	0.011	−0.073	−0.028	< 0.001
Neighborhood Median Income/ 50000	Marijuana Use	0.004	0.011	−0.017	0.025	0.698
Major Depressive Disorder	Marijuana Use	0.024	0.011	0.002	0.045	0.032
Suicide Attempt	Marijuana Use	0.064	0.014	0.037	0.092	< 0.001
Hopeless	Marijuana Use	0.031	0.015	0.001	0.060	0.041
Race/Ethnicity (Black)	Marijuana Use	−0.031	0.011	−0.054	−0.009	0.006
Race/Ethnicity (Latino)	Marijuana Use	−0.010	0.011	−0.031	0.011	0.334
Race/Ethnicity (Asian)	Marijuana Use	−0.019	0.009	−0.037	−0.001	0.039
Race/Ethnicity (Other)	Marijuana Use	−0.004	0.010	−0.023	0.015	0.655
Intercept	Marijuana Use	−0.686	0.261	−1.197	−0.174	0.009

**Table 2.** Summary of Structural Equation Model (SEM), By Gender

Independent Variable	Dependent Variable	B	SE	95%	CI	P
Females						
Age (Years)	Tobacco Use	0.084	0.013	0.058	0.110	< 0.001
Family Income	Tobacco Use	0.007	0.022	−0.036	0.050	0.754
Parental Education	Tobacco Use	−0.019	0.018	−0.055	0.017	0.307
Married Household	Tobacco Use	−0.084	0.016	−0.116	−0.052	< 0.001
Neighborhood Median Income/ 50000	Tobacco Use	0.034	0.016	0.003	0.065	0.032
Major Depressive Disorder	Tobacco Use	0.049	0.016	0.018	0.081	0.002
Suicide Attempt	Tobacco Use	0.104	0.020	0.065	0.142	< 0.001
Hopeless	Tobacco Use	0.087	0.022	0.045	0.130	< 0.001
Race/Ethnicity (Black)	Tobacco Use	−0.050	0.017	−0.082	−0.018	0.003
Race/Ethnicity (Latino)	Tobacco Use	−0.002	0.016	−0.033	0.029	0.893
Race/Ethnicity (Asian)	Tobacco Use	−0.035	0.014	−0.061	−0.008	0.010
Race/Ethnicity (Other)	Tobacco Use	0.007	0.014	−0.020	0.035	0.609
Intercept	Tobacco Use	−0.976	0.381	−1.724	−0.229	0.010


Age (Years)	Marijuana Use	0.071	0.013	0.045	0.098	< 0.001
Family Income	Marijuana Use	0.002	0.022	−0.041	0.046	0.911
Parental Education	Marijuana Use	−0.022	0.018	−0.058	0.014	0.225
Married Household	Marijuana Use	−0.089	0.016	−0.121	−0.056	< 0.001
Neighborhood Median Income/ 50000	Marijuana Use	−0.009	0.016	−0.040	0.022	0.562
Major Depressive Disorder	Marijuana Use	0.037	0.016	0.006	0.068	0.020
Suicide Attempt	Marijuana Use	0.127	0.019	0.090	0.165	< 0.001
Hopeless	Marijuana Use	0.042	0.021	0.000	0.083	0.051
Race/Ethnicity (Black)	Marijuana Use	−0.027	0.017	−0.059	0.005	0.104
Race/Ethnicity (Latino)	Marijuana Use	−0.006	0.016	−0.036	0.025	0.725
Race/Ethnicity (Asian)	Marijuana Use	−0.022	0.014	−0.048	0.005	0.106
Race/Ethnicity (Other)	Marijuana Use	0.006	0.014	−0.022	0.034	0.668
Intercept	Marijuana Use	−0.656	0.382	−1.404	0.092	0.086

Males						
Age (Years)	Tobacco Use	0.067	0.013	0.042	0.092	< 0.001
Family Income	Tobacco Use	−0.031	0.021	−0.072	0.010	0.134
Parental Education	Tobacco Use	−0.040	0.017	−0.073	−0.006	0.021
Married Household	Tobacco Use	−0.025	0.016	−0.056	0.006	0.116
Neighborhood Median Income/ 50000	Tobacco Use	0.007	0.015	−0.023	0.036	0.654
Major Depressive Disorder	Tobacco Use	0.015	0.015	−0.015	0.045	0.338
Suicide Attempt	Tobacco Use	0.023	0.021	−0.018	0.064	0.272
Hopeless	Tobacco Use	0.022	0.021	−0.020	0.063	0.306
Race/Ethnicity (Black)	Tobacco Use	−0.040	0.016	−0.071	−0.010	0.010
Race/Ethnicity (Latino)	Tobacco Use	0.002	0.015	−0.027	0.031	0.912
Race/Ethnicity (Asian)	Tobacco Use	−0.021	0.013	−0.046	0.004	0.101
Race/Ethnicity (Other)	Tobacco Use	0.025	0.013	−0.001	0.051	0.063
Intercept	Tobacco Use	−0.222	0.360	−0.928	0.483	0.537


Age (Years)	Marijuana Use	0.084	0.013	0.059	0.109	< 0.001
Family Income	Marijuana Use	−0.052	0.020	−0.092	−0.011	0.012
Parental Education	Marijuana Use	−0.030	0.017	−0.064	0.003	0.076
Married Household	Marijuana Use	−0.019	0.016	−0.050	0.012	0.223
Neighborhood Median Income/ 50000	Marijuana Use	0.015	0.015	−0.014	0.045	0.315
Major Depressive Disorder	Marijuana Use	0.015	0.015	−0.015	0.045	0.317
Suicide Attempt	Marijuana Use	0.004	0.020	−0.036	0.044	0.836
Hopeless	Marijuana Use	0.029	0.021	−0.012	0.070	0.166
Race/Ethnicity (Black)	Marijuana Use	−0.036	0.016	−0.067	−0.005	0.022
Race/Ethnicity (Latino)	Marijuana Use	−0.012	0.015	−0.041	0.017	0.414
Race/Ethnicity (Asian)	Marijuana Use	−0.018	0.013	−0.043	0.007	0.151
Race/Ethnicity (Other)	Marijuana Use	−0.013	0.013	−0.039	0.013	0.335
Intercept	Marijuana Use	−0.683	0.359	−1.388	0.021	0.057
